# Extra corporeal membrane oxygenation to facilitate lung protective ventilation and prevent ventilator-induced lung injury in severe Pneumocystis pneumonia with pneumomediastinum: a case report and short literature review

**DOI:** 10.1186/s12890-016-0214-4

**Published:** 2016-04-14

**Authors:** Husain Shabbir Ali, Ibrahim Fawzy Hassan, Saibu George

**Affiliations:** Department of Medical ICU, Hamad General Hospital, P.O. Box 3050, Doha, State of Qatar Qatar

**Keywords:** Extra corporeal membrane oxygenation, Pneumocystis jirovecii pneumonia, Human immunodeficiency virus, Pneumomediastinum

## Abstract

**Background:**

Pulmonary infections caused by Pneumocystis jirovecii in immunocompromised host can be associated with cysts, pneumatoceles and air leaks that can progress to pneumomediastinum and pneumothoraxes. In such cases, it can be challenging to maintain adequate gas exchange by conventional mechanical ventilation and at the same time prevent further ventilator-induced lung injury. We report a young HIV positive male with poorly compliant lungs and pneumomediastinum secondary to severe Pneumocystis infection, rescued with veno-venous extra corporeal membrane oxygenation (V-V ECMO).

**Case presentation:**

A 26 year old male with no significant past medical history was admitted with fever, cough and shortness of breath. He initially required non-invasive ventilation for respiratory failure. However, his respiratory function progressively deteriorated due to increasing pulmonary infiltrates and development of pneumomediastinum, eventually requiring endotracheal intubation and invasive ventilation. Despite attempts at optimizing gas exchange by ventilatory maneuvers, patients’ pulmonary parameters worsened necessitating rescue ECMO therapy. The introduction of V-V ECMO facilitated the use of ultra-protective lung ventilation and prevented progression of pneumomediastinum, maintaining optimal gas exchange. It allowed time for the antibiotics to show effect and pulmonary parenchyma to heal. Further diagnostic workup revealed Pneumocystis jirovecii as the causative organism for pneumonia and serology confirmed Human Immunodeficiency Virus infection. Patient was successfully treated with appropriate antimicrobials and de-cannulated after six days of ECMO support.

**Conclusion:**

ECMO was an effective salvage therapy in HIV positive patient with an otherwise fatal respiratory failure due to Pneumocystis pneumonia and air leak syndrome.

## Background

Lung protective ventilation and prevention of ventilator-induced lung injury (VILI) are a cornerstone in the management of Acute Respiratory Distress Syndrome (ARDS) [1]. But implementing these strategies in patients with poor lung compliance can lead to inadequate gas exchange. The use of veno-venous extra corporeal membrane oxygenation (V-V ECMO) to facilitate lung rest and maintain optimal gas exchange has been significantly growing in the past few years [2, 3].

Prognosis of the underlying disease, timing, quality of life of survivors and the possibility of being lung transplant candidate remain important considerations for patient selection. There are concerns about initiating ECMO support in immunocompromised patients especially those with underlying HIV (Human Immunodeficiency Virus) infection and AIDS (Acquired Immune Deficiency Syndrome) due to poor outcomes [4]. We report a case of life threatening respiratory failure due to severe Pneumocystis jirovecii infection complicated by pneumomediastinum in a HIV infected patient who was rescued by V-V ECMO.

## Case presentation

**Fig. 1:**
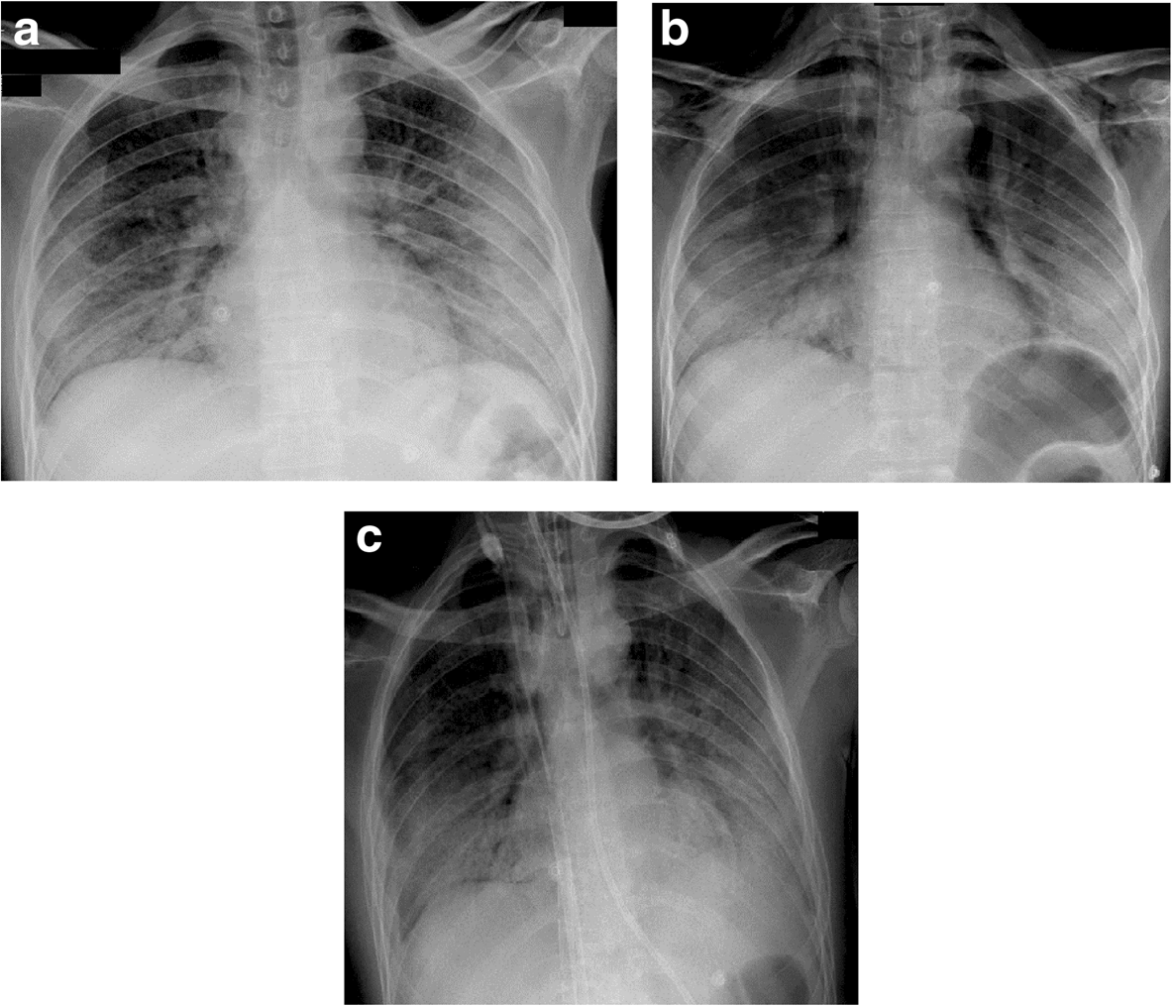
Chest x-ray of patient. **a** On admission: Bilateral middle and lower zone air space opacities with air bronchograms; **b** After application of non-invasive ventilation: Subcutaneous emphysema around neck and axillary region, hyper-lucency around mediastinal structures suggestive of pneumomediastinum and bilateral pulmonary infiltrates. **c** Post endotracheal intubation and ECMO cannulation

A 26 year old male with no significant past medical history presented to the emergency department with complaints of fever, dry cough, difficulty in breathing and chest pain of 15 days duration. Initial assessment showed an averagely built male with body mass index (BMI) 18.8 kg/m^2^ (normal: 18.5-24.9 kg/m^2^), fever (38.9 °C), tachycardia (heart rate 120/min, regular), tachypnea (respiratory rate 35/min), hypoxia (pulse oximetry: 85 % on room air, PaO_2_: 55 mm Hg) and normal blood pressure (126/76 mm Hg). Systemic examination was unremarkable other than reduced air entry at both lung bases. Blood investigations revealed an elevated white cell count of 14.2 x 10^9^/L (normal: 4.5-10 x 10^9^/L), predominantly neutrophilic; serum electrolytes, renal function, liver function, coagulation profile were within normal limits; lactate was 2.2 mmol/L (normal: <2 mmol/L) and procalcitonin was 0.6 ng/mL (normal: <0.5 ng/mL). Blood cultures, sputum for gram’s stain and nasal swab for viral polymerase chain reaction (PCR) were collected. Chest x-ray showed bilateral middle and lower zone air space opacities (Fig. 1a). After oxygen supplementation via nasal cannula, fluid resuscitation and empirical antimicrobial therapy (ceftriaxone 2 gm i.v. once daily, azithromycin 500 mg i.v. once daily and oseltamivir 75 mg orally twice daily) patient was stabilized (PaO_2_ 80 mm Hg on oxygen 2 L/min through nasal cannula) and admitted to medical ward.

**Fig. 2:**
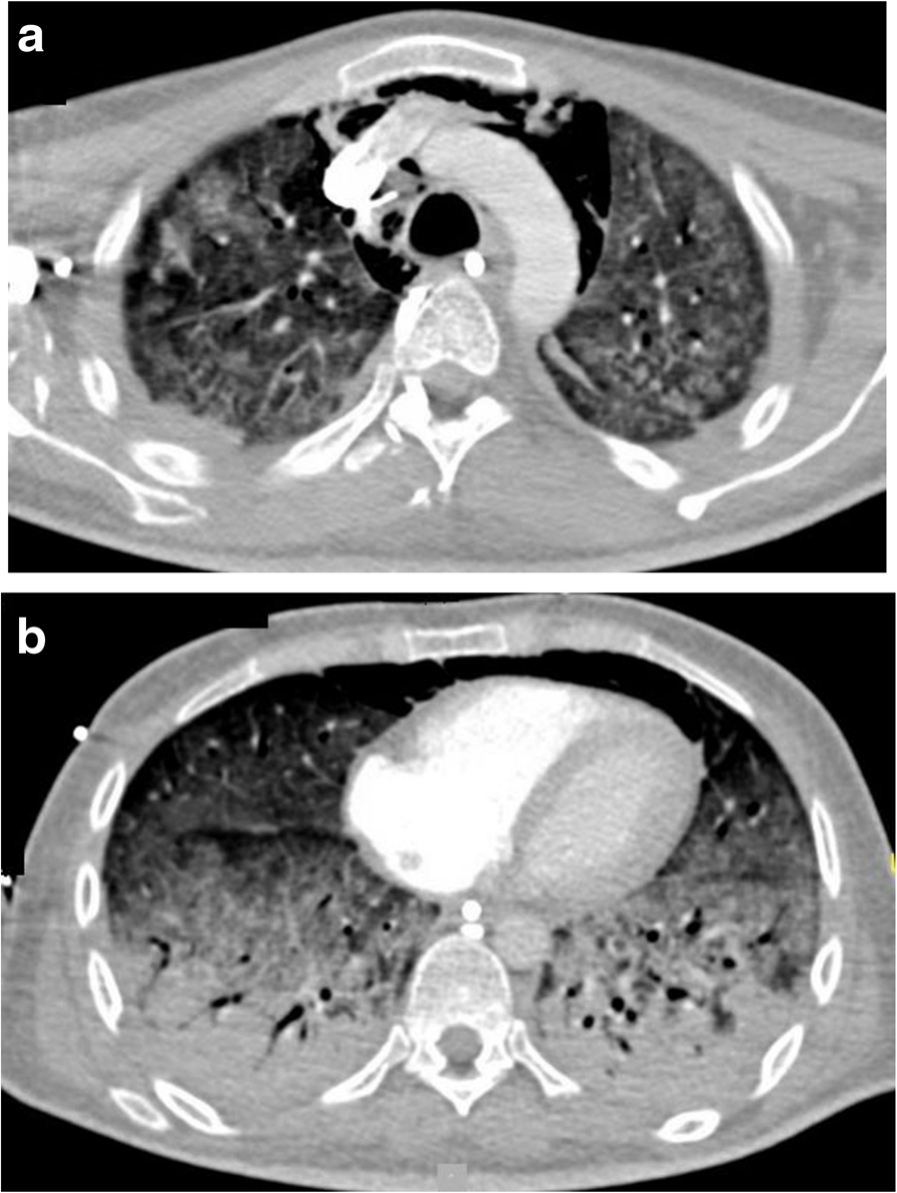
CT scan of patient. **a** At the level of arch of aorta. **b** Lung bases. Shows bilateral diffuse ground glass opacity with extensive basal consolidation. Pneumomediastinum: air in the anterior and superior mediastinum

Due to worsening of respiratory function (respiratory rate 30/min and PaO_2_ 60 mm Hg on supplemental oxygen 5 L/min through face mask) on the 2^nd^ day post hospitalization, patient was transferred to medical intensive care unit (MICU). Non-invasive ventilation (NIV) via face mask was initiated to improve respiratory parameters and ceftriaxone was escalated to piperacillin/tazobactam 4.5 gm i.v. every 6 h; azithromycin and oseltamivir were continued. Blood and respiratory samples collected initially did not reveal causative organism for pneumonia. Over the next 2 days patient’s condition remained steady with single organ (respiratory) failure requiring intermittent NIV (averaging 16 h/day) with oxygen supplement (FiO_2_ 0.5). As the patient was neurologically appropriate and understood his clinical condition, good adherence to NIV was maintained. On Day 4 of ICU admission, patient’s respiratory function further deteriorated (respiratory rate 40/min; PaO_2_ 50 mm Hg on FiO_2_ 0.6) and chest x-ray showed subcutaneous emphysema with pneumomediastinum in addition to bilateral pulmonary infiltrates (Fig. 1b). After detailed discussion with the patient and addressing his concerns, he was intubated and mechanical ventilation was initiated. This was followed by diagnostic bronchoscopy with broncho-alveolar lavage (BAL) fluid collection and CT (computerized tomography) scan of chest which revealed bilateral diffuse ground glass opacity with extensive basal consolidation and pneumomediastinum (Fig. 2). Bedside trans-thoracic echocardiography showed good left ventricular function and non-dilated right ventricle. Since serial imaging did not show worsening of pneumomediastinum, it was managed conservatively. Despite optimal sedation, analgesia and neuro-muscular paralysis it was not possible to achieve adequate gas exchange with lung protective ventilation and hence, ‘Severe Respiratory Failure’ team at our institution was consulted.

On evaluation by the Severe Respiratory Failure team (Table 1), tidal volume was decreasing to critical levels (225 mL) trying to achieve safe ventilation pressures, PaO_2_/FiO_2_ was 200 mm Hg on 45 % of FiO_2_ and 10 cm H_2_O of Positive End-Expiratory Pressure (PEEP). As well patient had severe respiratory acidosis with PaCO_2_ of 109 mm Hg and pH 7.01; pulmonary compliance was only 9 ml/cm H_2_O and Murray score was 3. The decision was made to place the patient on V-V ECMO to facilitate lung protective ventilation, prevent progression of pneumomediastinum and improve respiratory acidosis. 25 French drainage cannula was inserted percutaneously into inferior vena cava via left femoral approach and 23 French return cannula was inserted into the inferior vena cava via right femoral approach under fluoroscopy guidance. Post cannulation, ECMO blood flow and sweep flow were titrated to 4 liters/min each in order to achieve optimal gas exchange. Mechanical ventilation was switched to ultra-protective strategy using Pressure Control Ventilation (PCV) with Peak Inspiratory Pressure (PIP) 15 cm H_2_O, PEEP 5 cm H_2_O, respiratory rate 10/min and FiO_2_ 21 %. Patient was anticoagulated with intravenous heparin infusion targeting an APTT of 50-70 s. Cotrimoxazole 250 mg i.v. every 6 h (20 mg/kg/day of Trimethoprim) and prednisone 40 mg twice daily through feeding tube were started after Pneumocystis jirovecii was identified in the broncho-alveolar lavage (BAL) fluid on ECMO day 1. Subsequently, serology confirmed HIV-1 infection with CD4+ cell count of 84/mm^3^ of blood (normal: 600-1500/mm^3^ of blood) and HIV viral load of 907,302 copies/mL. Patient’s respiratory parameters progressively improved (Table 1), his blood pressure was maintained without vasopressor support and other organs function including the kidneys remained normal throughout ECMO therapy and ICU stay. Neuro-muscular paralysis was held on ECMO day 2 followed by weaning off sedation on ECMO day 3. The patient was able to tolerate pressure support ventilation (PSV) by ECMO day 4 and he was successfully de-cannulated after 6 days of V-V ECMO support. Patient was extubated the next day, followed by aggressive physiotherapy/mobilization and transfer to medical ward in the next 48 h. There were no complications related to ECMO therapy in this patient during ICU stay and on follow-up post discharge. Patient was explained about his diagnosis and highly active anti-retroviral therapy (HAART: Efavirenz, Emtricitabine and Tenofovir) was initiated in the medical ward. He was discharged in good general condition after 21 days of hospitalization with follow-up appointment in Infectious Disease clinic.

**Table 1:** Ventilator and ECMO parameters

	Pre-Intubation	Post-Intubation	ECMO Day 1	ECMO Day 2	ECMO Day 3	ECMO Day 4	ECMO Day 5	ECMO Day 6
Mode of Ventilation	PSV (NIV)	PCV	PCV	PCV	PCV	PSV	PSV	PSV
RR	40	35	10	10	10	25	25	22
PIP	16	35	15	15	15	13	11	11
PEEP	4	10	5	5	5	3	3	3
VT	-	225	90	200	400	400	360	370
FiO_2_	40 %	45 %	21 %	21 %	21 %	30 %	30 %	30 %
P/F ratio	-	200	-	-	-	-	-	-
Blood Flow	-	-	4	3.78	3.75	3.7	3.6	3.6
Sweep Flow	-	-	4	2.5	1.0	0.5	0	0
pH	7.46	7.01	7.44	7.39	7.42	7.47	7.5	7.5
PaO_2_	73	90	83	161	114	70	74	86
PaCO_2_	44	109	49	43	45	38	33	35

*RR* Respiratory rate, *PIP* Peak Inspiratory Pressure (cm H_2_O), *PEEP* Positive End Expiratory Pressure (cm H_2_O), *VT* Tidal volume (mL), *FiO*_*2*_ Fraction of inspired oxygen (%), *P/F ratio* Ratio of partial pressure arterial oxygen and fraction of inspired oxygen (mm Hg), *PaO*_*2*_ Partial pressure of oxygen in arterial blood (mm Hg), *PaCO*_*2*_ Partial pressure of carbon dioxide in arterial blood (mm Hg), *NIV* Non Invasive Ventilation, *PSV* Pressure Support Ventilation, *PCV* Pressure Control Ventilation

## Discussion

The frequency of ICU admission for Pneumocystis pneumonia in immunocompromised patients is decreasing but mortality is high if it progresses to respiratory failure requiring mechanical ventilation [5]. The most common manifestation on chest radiographs is bilateral interstitial or alveolar opacities. Parenchymal/sub-pleural cysts and pneumatoceles are frequently seen on CT scan. Spontaneous pneumothorax can occur in up to 36 % of patients with Pneumocystis jirovecii pneumonia [5, 6]. However, spontaneous pneumomediastinum is an uncommon complication with no reported incidence rates. The mechanism of spontaneous pneumomediastinum can be explained by the existence of a decreasing pressure gradient between the alveoli and the lung interstitium that can result in alveolar rupture. This leads to the accumulation of air in the interstitium that circulates centripetally through the venous sheaths to the hilum and mediastinum as the pressure in the mediastinum is lower than that of the lung periphery [7]. Our patient developed pneumomediastinum while on NIV. Barotrauma is very uncommon and pneumothorax following NIV occurs in less than 5 % of cases [8]. Development of pneumomediastinum in Pneumocystis jirovecii pneumonia patient receiving NIV has been reported previously [9]. This could be explained by the rupture of pre-existing pneumatoceles or cysts under the effect of positive pressure ventilation.

Acute respiratory distress syndrome (ARDS) is a life threatening respiratory condition. The Berlin definition classifies ARDS as mild, moderate and severe according to the value of PaO_2_/FiO_2_ ratio (Table 2) [10]. The main supportive therapy for ARDS is positive pressure mechanical ventilation which helps to ensure adequate oxygenation. A landmark trial conducted in the late 1990s by the ARDS Network compared conventional tidal volume of 12 ml/kg with low tidal volume of 6 ml/kg and permissive hypercapnia. A 9 % absolute mortality reduction was found in the low tidal volume ventilation group. In this study, target tidal volume was calculated based on ideal body weight (IBW) with targeted plateau pressures of <30 cm H_2_O and permissive hypercapnia [1]. Our patient did not retain CO_2_ while breathing spontaneously or supported by NIV. But soon after endotracheal intubation and mechanical ventilation he developed severe hypercapnia. This could be explained by the fact that during spontaneous breathing patient was inhaling large tidal volumes which resulted in increased minute ventilation and adequate CO_2_ clearance at the expense of high trans-pulmonary pressures resulting in worsening of air leak. After intubation, sedation and paralysis; lung protective ventilation was provided which resulted in severe (permissive) hypercapnia. Thus, ventilating this patient with ARDS and air leak syndrome was challenging because a balance was to be maintained between providing adequate gas exchange and preventing worsening of pneumomediastinum. The Extracorporeal Life Support Organization (ELSO) guidelines for Adult Respiratory Failure 2013 have listed carbon dioxide retention on mechanical ventilation despite high plateau pressures (>30 cm H_2_O) and severe air leak syndrome as indications for initiating extracorporeal life support [11]. Based on these recommendations we provided ECMO therapy to our patient, as a bridge to recovery from severe pulmonary infection.

**Table 2:** ARDS Berlin definition

Timing:	Within 1 week of a known clinical insult or new or worsening respiratory symptoms.
Chest imaging^a^:	Bilateral opacities — not fully explained by effusions, lobar/lung collapse, or nodules.
Origin of edema:	Respiratory failure not fully explained by cardiac failure or fluid overload.
	Need objective assessment (e.g., echocardiography) to exclude hydrostatic edema if no risk factor present
Oxygenation^b^:	
• Mild - 200 mm Hg < PaO_2_/FiO_2_ ≤ 300 mm Hg with PEEP or CPAP ≥5 cm H_2_O^c^	
• Moderate - 100 mm Hg < PaO_2_/FiO_2_ ≤ 200 mm Hg with PEEP ≥5 cm H_2_O	
• Severe - PaO_2_/FiO_2_ ≤ 100 mm Hg with PEEP ≥5 cm H_2_O	

*CPAP* continuous positive airway pressure, *FiO*_*2*_ fraction of inspired oxygen, *PaO*_*2*_ partial pressure of arterial oxygen, *PEEP* positive end-expiratory pressure

^a^Chest radiograph or computed tomography scan

^b^If altitude is higher than 1000 m, the correction factor should be calculated as follows: [PaO_2_/FIO_2_*(barometric pressure/760)]

^c^This may be delivered noninvasively in the mild acute respiratory distress syndrome group

There are very few case reports of ECMO therapy for severe respiratory failure due to Pneumocystis pneumonia in HIV/AIDS patients (Table 3) [9, 12–15]. All reported patients required V-V ECMO support for primarily respiratory failure other than the case reported by Gutermann et al, who was provided V-A (veno-arterial) ECMO therapy post cardiac arrest. 5 out of 8 patients had concomitant pneumothorax/pneumomediastinum which could have possibly worsened the respiratory failure, necessitating rescue ECMO therapy. The timing for initiation of V-V ECMO in severe respiratory failure remains debatable. However, based on previous studies it has been observed that increased pre-ECMO ventilation duration is associated with worse outcomes [16]. One of the exclusion criteria in CESAR trial was high pressure (>30 cm H_2_O of peak inspiratory pressure) and/or high FiO_2_ (>0.8) ventilation >7 days [2]. In our patient the duration of pre-ECMO invasive ventilation was very short and this could be one of the contributing factor for his improved outcome. Fifty percent of the reported cases survived to hospital discharge. It is interesting to note that the patients who survived had a shorter duration of ECMO support (mean 9.25 versus 41 days). Advanced patient age, increased pre-ECMO ventilation duration, diagnosis category and complications while on ECMO are some of the factors associated with adverse outcomes in patients receiving ECMO support [17].

**Table 3:** Adult patients with HIV/AIDS and severe Pneumocystis jirovecii pneumonia requiring ECMO therapy

Patient (Ref)	Age (years)/Gender (M/F)	Pre-ECMO invasive ventilation (days)	Pneumothorax/Pneumomediastinum	Pre-ECMO P/F ratio; PaCO_2_; pH	ECMO Configuration	Duration of ECMO (days)	CD4 count (cells/mm^3^)	HIV viral load (copies/mL)	Anti-Pneumocystis treatment	Timing of ART initiation	Outcome
Gutermann et al [10]	55/M	4	No	NR; NR; NR	Veno-arterial	4	9	80,235	TMP/SMX	Post-ECMO	Survived to hospital discharge
Steppan [11]	39/M	8	Yes	NR; NR; NR	Veno-venous	14	69	6297	CLI + PI, then ATQ, then TMP/SMX	Pre-ECMO	Died on ECMO
Goodman et al [12]	25/M	NR	No	63.6; 52.9; 7.38	Veno-venous	69	36	622,234	PI, then CLI + PQ, then TMP/SMX	Pre-ECMO	Died on ECMO
Goodman et al [12]	30/F	3	Yes	50.1; 41.6; 7.39	Veno-venous	7	13	976,631	TMP/SMX	Post-ECMO	Survived to hospital discharge
De Rosa et al [13]	21/F	NR	Yes	120; NR; NR	Veno-venous	20	2	118,330	TMP/SMX, then CLI + PQ	NR	Survived to hospital discharge
De Rosa et al [13]	24/M	NR	No	100; NR; NR	Veno-venous	24	3	50,728	TMP/SMX, then CLI + PQ + ATQ	During ECMO	Died in hospital post ECMO
Cawcutt et al [14]	45/M	NR	Yes	50; NR; NR	Veno-venous	57	33	113,000	TMP/SMX, CLI, PQ	Pre-ECMO	Died in hospital post ECMO
Our patient	26/M	1	Yes	200; 109; 7.01	Veno-venous	6	84	907,302	TMP/SMX	Post-ECMO	Survived to hospital discharge

*P/F ratio* Ratio of partial pressure arterial oxygen and fraction of inspired oxygen (mm Hg), *PaCO*_2_ Partial pressure of carbon dioxide in arterial blood (mm Hg), *ART* Antiretroviral therapy, *TMP* Trimethoprim, *SMX* Sulfamethoxazole, *CLI* Clindamycin, *PI* Pentamidine, *ATQ* Atovaquone, *PQ* Primaquine, *NR* Not reported

Patients in need of V-V ECMO support have a high predicted mortality, and implementing an invasive therapy that requires central venous cannulation, systemic anticoagulation, and exposure to an extracorporeal bypass circuit involves substantial risk. The most common complication encountered in ECMO patients is bleeding. Cannula site, surgical site, central nervous system and gastrointestinal bleeding are the most frequent hemorrhagic complications. Infection is also relatively common, with culture proven infections occurring in approximately 20 % of adult respiratory ECMO patients. Mechanical complications such as oxygenator failure, clotting, air in the circuit and tubing rupture are other important considerations [18, 19].

In the CESAR trial [2], not all patients transferred to the ECMO center received ECMO therapy. Despite this their outcomes were better than those managed in conventional treatment centers. This highlights the fact that management of patients with severe respiratory failure by a specialized team in units that have an ECMO-based management protocol improves outcome. As such, we have developed a ‘Severe Respiratory Failure’ team at our hospital that receives referrals from ICUs across the country. Their role is to evaluate and manage patients with life threatening respiratory failure who fail conventional ventilation.

## Conclusion

V-V ECMO was an effective rescue therapy in HIV positive patient with an otherwise fatal respiratory failure due to Pneumocystis pneumonia and air leak syndrome. This case report emphasizes on the fact that the use of ECMO support in HIV/AIDS patient is not always futile and can be considered if the patient has favorable baseline characteristics and the primary problem is respiratory failure that has a reversible etiology. It also highlights the role of ECMO in allowing ultra-protective lung ventilation and preventing ventilator-induced lung injury in patients with poorly compliant lungs.

### Ethics approval

The ‘Medical Research Center’ at Hamad Medical Corporation, Qatar has granted permission for publication of this case report.

### Consent for publication

Written informed consent was obtained from the patient for publication of this case report and any accompanying images. A copy of the written consent is available for review by the Editor-in-Chief of this journal.

### Availability of data

Data underlying the conclusions drawn is contained in the manuscript and its supporting files.

## Abbreviations

AIDS: acquired immune deficiency syndrome
APTT: activated partial thromboplastin time
ARDS: acute respiratory distress syndrome
BAL: broncho-alveolar lavage
BMI: body mass index
CT: computerized tomography
ELSO: extra-corporeal life support organization
FiO_2_: fraction of inspired oxygen
HIV: human immunodeficiency virus
MICU: medical intensive care unit
NIV: non-invasive ventilation
PaCO_2_: partial pressure of carbon dioxide in arterial blood
PaO_2_: partial pressure of oxygen in arterial blood
PCR: polymerase chain reaction
PCV: pressure control ventilation
PEEP: positive end expiratory pressure
PIP: peak inspiratory pressure
PSV: pressure support ventilation
VILI: ventilator-induced lung injury
V-V ECMO: veno-venous extra corporeal membrane oxygenation
